# Patient Race Independently Predicts Timeliness of Breast Cancer Reconstructive Care

**DOI:** 10.3390/jcm14186532

**Published:** 2025-09-17

**Authors:** Jacquelyn Roth, Bernice Z. Yu, Maxwell Godek, Ethan Fung, Brooke Barrow, Peter J. Taub, Peter W. Henderson

**Affiliations:** Icahn School of Medicine at Mount Sinai Hospital, New York, NY 10029, USA; jacquelyn.roth@mountsinai.org (J.R.); bernice.yu@mountsinai.org (B.Z.Y.); max.godek@mountsinai.org (M.G.); ethan.fung@mountsinai.org (E.F.); brooke.barrow@mountsinai.org (B.B.); peter.taub@mountsinai.org (P.J.T.)

**Keywords:** breast reconstruction, social determinants of health, racial disparities, care delays, clinical timelines

## Abstract

**Background/Objectives:** While higher complication rates among minority patients following breast reconstruction are documented, the role of lengthier care intervals in perpetuating these disparities remains unclear. This study assesses whether race influences the timeliness of breast reconstructive care for patients following breast cancer diagnoses. **Methods:** A retrospective analysis of breast reconstruction patients between 2017 and 2023 was conducted. Primary outcomes comprised intervals from breast cancer diagnosis to plastic and reconstructive surgery (PRS) consultation, index reconstructive procedure, and final reconstructive procedure. Regression models assessed the impact of race on outcomes. **Results:** Of the 1662 patients included, 745 identified as White (44.8%), 337 as Black (20.3%), 199 as Asian (12.0%), 278 as “Other” races (16.7%), and 103 did not specify race (6.2%). Baseline characteristics differed significantly across groups (*p* < 0.001). Latino patients comprised the majority of the ‘Other’ (50%) and ‘Unknown’ (75%) race categories. On multivariable regression, intervals to PRS consultation and index procedure were significantly prolonged for Black (β = 0.307, *p* = 0.001 and β = 0.254, *p* < 0.001, respectively) and “Other” race (β = 0.332, *p* = 0.006 and β = 0.283, *p* = 0.001) patients, while Black patients also faced significantly longer intervals to the final procedure (β = 0.213, *p* = 0.001). **Conclusions:** Prolonged care intervals for non-White patients persist at multiple stages of breast reconstruction, potentially exacerbating outcome disparities. The present study implicates logistical barriers such as fragmented care, scheduling, and transportation challenges, as well as provider- or patient-level bias, as contributors to race-based disparities in timely care.

## 1. Introduction

As awareness grows around social determinants of health, mounting evidence highlights race-based inequities within the U.S. healthcare system [1,2,3,4,5]. Numerous studies demonstrate that Black and Hispanic patients receive fewer surgical interventions and lower-quality care compared to their White counterparts [6,7,8,9,10,11,12,13,14,15,16,17,18,19,20]. Moreover, reduced access to preventive care and screening, delayed symptom recognition, lower health literacy, underinsurance, and geographic barriers to specialized services contribute to limited preoperative care, leading to more advanced disease presentation among minority patients [21]. In addition, minority patients tend to be treated at lower-volume hospitals or by less experienced surgeons [22,23,24,25,26]. Consequently, non-White patients experience higher postoperative complication rates [27,28] increased surgery-related mortality, [29,30,31,32,33,34,35,36,37,38,39,40,41] and a greater risk of disease recurrence [42,43].

Breast reconstruction following mastectomy is no exception to race-based disparities. Despite evidence of superior psychosocial and sexual well-being benefits from reconstruction [39], prior studies indicate that Black women experience worse clinical outcomes than White women, including longer hospital stays and higher complication rates [44]. Disparities are also apparent earlier in the care process, with utilization rates significantly lower among minority populations [45,46]. These utilization gaps have persisted even after the 1998 Women’s Health and Cancer Rights Act (WHCRA) mandated insurance coverage [45,46,47,48,49], underscoring the role of structural, cultural, and systemic factors beyond insurance access.

Given the persistent race-based utilization and outcome disparities observed in the literature, additional contributing factors warrant further investigation. In particular, although guidelines recommend plastic surgery referrals at the time of diagnosis [50], emphasizing the importance of timely intervention for optimal psychosocial and sexual health outcomes [51], research quantifying the timeliness of breast reconstructive care remains limited. As such, the objective of this study is to examine the impact of patient race on the timing of breast reconstructive care following breast cancer diagnosis to identify factors driving these disparities and to inform targeted interventions.

## 2. Materials and Methods

### 2.1. Ethical Review

The following study was approved by the Institutional Review Board at Mount Sinai Hospital (IRB 20-01356). All patient data were deidentified and analyzed accordingly.

### 2.2. Study Design and Inclusion/Exclusion Criteria

A retrospective analysis was performed on breast cancer patients who underwent primary breast reconstruction at a single institution from 2017 to 2023. The study included patients who had either immediate or delayed implant-based or autologous reconstruction. Patients with incomplete or missing data were excluded from the analysis.

### 2.3. Patient Data Collection

Patients were classified by self-identified race as indicated on patient intake surveys. Races included Black, White, Asian and Other. Patients who chose not to specify Race were classified as Unknown. Throughout this study, the term “non-White” refers to individuals identifying as Black, Asian, Other, or Unknown. Data was also collected on patient age, ethnicity, body mass index, and baseline health comorbidities including diabetes, hypertension (HTN), hyperlipidemia (HLD), asthma, coronary artery disease (CAD), prior myocardial infarction (MI), heart failure, peripheral artery disease (PAD), cerebrovascular accidents (CVA), chronic obstructive pulmonary disease (COPD), liver disease and renal disease.

Reconstruction types were categorized as immediate autologous, direct-to-implant (DTI), delayed autologous, delayed implant, or “other”. Delayed reconstruction included patients who underwent primary closure or tissue expander-based procedures at the time of mastectomy, followed by definitive implant-based or autologous reconstruction at a later date. Patients receiving a combination of implant-based and autologous reconstruction were grouped under the “other” category.

The primary outcome measures were the intervals from breast cancer diagnosis, defined by the date of core needle biopsy, to key milestones in the reconstructive process. These included the initial consultation with plastic and reconstructive surgery (PRS), the initiation of reconstruction marked by the index operation, and the completion of reconstruction marked by the final operation. Analysis was conducted on the entire cohort as well as an autologous breast reconstruction (ABR) subgroup and an implant-based reconstruction (IBR) subgroup.

### 2.4. Statistical Analyses

Continuous variables were described using median and range while categorical variables were presented as counts and percentages. Kolmogorov–Smirnov and Shapiro–Wilk tests assessed normality of the data; Pearson’s Chi-squared test and analysis of variance (ANOVA) were used for normally distributed data and Fisher’s exact test and the Kruskal–Wallis test for non-normally distributed data. Univariable and multivariable log-time-adjusted linear regressions evaluated the impact of age on quantitative outcomes, with results given as standardized beta coefficients (β). To facilitate these analyses, race was treated as a categorical predictor by generating dummy variables for each non-White race category, using White patients as the reference group. Multivariable linear regression covariates included age, ethnicity, BMI, insurance type, comorbidities, and procedure type (including timing and laterality).

Statistical analysis was conducted using R Studio, and tables were created using the summary package (R Core Team, 2021; Sjoberg, 2021).

A *p*-value of <0.05 was considered statistically significant.

## 3. Results

The study included 1662 patients undergoing breast reconstruction (Table 1), including 745 patients who identified as White (44.8%), 337 patients who identified as Black (20.3%), 199 patients who identified as Asian (12.0%), 278 patients who identified as Other races (16.7%), and 103 patients who did not specify race (i.e., “Unknown”, 6.2%).

### 3.1. Baseline Characteristics

Significant differences were apparent across demographic and clinical characteristics of racial groups. Age (*p* < 0.001) and BMI (*p* < 0.001) were significantly different, with both lowest among Asian patients (Age: 48 ± 10 years, BMI: 24.6 ± 4.3) and highest among Black individuals (Age: 54 ± 11 years, BMI: 30.2 ± 5.8). Ethnicity varied significantly (*p* < 0.001), with the “Other” (50%) and “Unknown” (75%) groups having the highest proportion of Latino individuals, while White patients, Black patients and Asian patients were predominantly (>97%) non-Latino. Insurance status also varied (*p* < 0.001) with Medicare coverage highest among Black patients (31%) and Private insurance among White patients (68%).

Baseline health similarly differed between groups, with heart failure (*p* = 0.003), renal disease (*p* < 0.001), HTN (*p* < 0.001), HLD (*p* < 0.001), and diabetes (*p* < 0.001) all highest among Black patients. Conversely, the latter three, as well as CAD (*p* = 0.045), PAD (*p* = 0.028) and asthma (*p* < 0.001), were lowest among Asian patients.

Reconstruction type (*p* < 0.001) and laterality (*p* < 0.001) differed significantly, with unilateral procedures being more common in Black (54%) and Asian (59%) patients compared to White patients (34%), who had the highest proportion of bilateral procedures (66%).

Procedure times were significantly different between groups for both index and aggregate procedures (*p* < 0.001). The “Unknown” group had the longest times for both the index (7.18 ± 2.70 h) and aggregate procedures (7.94 ± 2.65 h), while White patients had the shortest lengths of both the index (5.06 ± 2.54 h) and aggregate procedures (6.16 ± 2.47 h).

Clinical timelines varied significantly across groups (*p* < 0.001). Asian individuals had the longest intervals between all stages, including from diagnosis to PRS consult (346 ± 3243 days), diagnosis to index procedure (382 ± 3174 days), and diagnosis to final procedure (473 ± 3209 days). In contrast, the shortest interval from diagnosis to PRS consult was observed for White patients (80 ± 233 days), while the shortest intervals to index (150 ± 210 days) and final procedures (280 ± 372 days) were observed for Black patients (*p* = 0.017).

### 3.2. Univariable Linear Regression Analysis

Univariable regression analysis (Table 2) revealed significant racial disparities in intervals between diagnosis and key milestones in the breast reconstruction process. Compared to White patients, all non-White patient cohorts experienced significantly longer intervals to PRS consultation and to index procedure. Among these groups, analysis suggested intervals were most extended among “Other” race patients (PRS consultation: β = 0.380, 95% CI: 0.188 to 0.571, *p* < 0.001; index procedure: β = 0.308, 95% CI: 0.164 to 0.451, *p* < 0.001), followed by Black patients (PRS consultation: β = 0.339, 95% CI: 0.162 to 0.516, *p* < 0.001; index procedure: β = 0.302, 95% CI: 0.168 to 0.435, *p* < 0.001), Asian patients (PRS consultation: β = 0.300, 95% CI: 0.083 to 0.517, *p* = 0.007; index procedure: β = 0.164, 95% CI: 0.001 to 0.326, *p* = 0.049) and those in the Unknown race category (PRS consultation: β = 0.296, 95% CI: 0.011 to 0.582, *p* = 0.042; index procedure: β = 0.297, 95% CI: 0.080 to 0.514, *p* = 0.007). Patients in the “Other” race group also showed longer intervals to the final procedure (β = 0.172, 95% CI: 0.005 to 0.339, *p* = 0.044).

Within the ABR cohort, Black patients (β = 0.238, 95% CI: 0.009 to 0.468, *p* = 0.042) and Asian patients (β = 0.320, 95% CI: 0.041 to 0.599, *p* = 0.025) experienced significantly longer intervals from diagnosis to PRS consultation, while patients in the “Other” racial category had significantly prolonged intervals from diagnosis to index procedure (β = 0.197, 95% CI: 0.003 to 0.392, *p* = 0.047).

In the IBR cohort, Black patients had significantly prolonged intervals across all phases, including from diagnosis to PRS consultation (β = 0.537, 95% CI: 0.242 to 0.831, *p* < 0.001), index procedure (β = 0.516, 95% CI: 0.298 to 0.734, *p* < 0.001), and final procedure (β = 0.342, 95% CI: 0.119 to 0.565, *p* = 0.003). Patients in the “Other” racial category also had significantly prolonged intervals from diagnosis to PRS consultation (β = 0.505, 95% CI: 0.226 to 0.785, *p* < 0.001), index procedure (β = 0.391, 95% CI: 0.180 to 0.603, *p* < 0.001), and final procedure (β = 0.254, 95% CI: 0.046 to 0.462, *p* = 0.017).

### 3.3. Multivariable Linear Regression Analysis

On multivariable regression (Table 3), Black patients continued to experience significantly prolonged intervals from diagnosis to PRS consultation (β = 0.307, 95% CI: 0.121 to 0.493, *p* = 0.001), index procedure (β = 0.254, 95% CI: 0.117 to 0.390, *p* < 0.001), and final procedure (β = 0.213, 95% CI: 0.084 to 0.342, *p* = 0.001). Patients in the “Other” racial category also continued to have significantly longer intervals from diagnosis to PRS consultation (β = 0.332, 95% CI: 0.095 to 0.568, *p* = 0.006) and index procedure (β = 0.283, 95% CI: 0.111 to 0.456, *p* = 0.001). Meanwhile, prolonged intervals only persisted between diagnosis and PRS consult for Asian patients (β = 0.267, 95% CI: 0.042 to 0.491, *p* = 0.020) and from diagnosis to index procedure for patients in the Unknown race category (β = 0.277, 95% CI: 0.011 to 0.544, *p* = 0.042).

Within the ABR cohort, Black patients (β = 0.256, 95% CI: 0.015 to 0.496, *p* = 0.037) and Asian patients (β = 0.299, 95% CI: 0.005 to 0.593, *p* = 0.046) both maintained significantly prolonged intervals between diagnosis and PRS consultation, but no significant differences were observed between diagnosis and index or final procedure.

In the IBR cohort, Black patients continued to experience significantly prolonged intervals between diagnosis and PRS consultation (β = 0.459, 95% CI: 0.148 to 0.770, *p* = 0.004), index procedure (β = 0.447, 95% CI: 0.218 to 0.675, *p* < 0.001), and final procedure (β = 0.322, 95% CI: 0.120 to 0.523, *p* = 0.002). Patients in the “Other” racial category also had significantly prolonged intervals from diagnosis to PRS consultation (β = 0.521, 95% CI: 0.141 to 0.900, *p* = 0.007) and index procedure (β = 0.411, 95% CI: 0.124 to 0.698, *p* = 0.005).

## 4. Discussion

The present study revealed prolonged care intervals across multiple stages of the breast cancer reconstruction process for non-White patients. These findings complement existing literature by identifying significant racial disparities within a previously unexplored aspect of reconstructive care. Moreover, findings of this study suggest that recommendations for PRS referrals at the time of diagnosis [50], intended to mitigate the psychological and physical burdens of mastectomy [51], are not being equitably implemented. As such, this study supports the integration of equity-focused initiatives such as standardized referral protocols, implicit bias training, and early involvement of social work to ensure timely, inclusive, and patient-centered reconstructive care.

While disparities were most consistent among Black and “Other” race patients, all non-White groups examined, including Asian and “Unknown” race patients, experienced delays at one or more key stages of the reconstructive process compared to White patients on multivariable analysis. Notably, while the specific racial identities of patients in the “Other” and “Unknown” categories are not defined, these groups are predominantly Latino (50% and 75%, respectively), supporting their classification as minority populations for analysis. These results align with previous research demonstrating that minority patients experience longer delays in accessing general surgical care compared to White patients [49]. Moreover, existing literature demonstrates these delays persist after controlling for insurance coverage and disease severity [52], and within healthcare systems designed to minimize disparities, such as the Veterans Health Administration [53], suggesting that systemic factors beyond financial constraints and individual healthcare systems, such as provider bias and logistical barriers, may play a role in prolonging clinical timelines for minority patients.

Logistical challenges previously shown to disproportionately affect minority patients include longer in-office wait times, inconvenient office hours, and transportation difficulties [4,54,55,56,57,58]. While such structural barriers can lead to missed or delayed care opportunities, further exacerbating disparities in surgical outcomes, these often reflect the interaction of broader socioeconomic and circumstantial variables that are external to the healthcare network. Beyond logistical challenges, provider biases may contribute to observed delays, with previous studies showing that racial and ethnic biases can contribute to differences in the urgency with which care is provided [59,60]. For example, Black race has been identified as a negative predictor for specialist consultation and treatment, limiting access to high-quality surgical care [15].

Given the persistence of outcome disparities [44] and the critical importance of timely care [51], the delays identified in this study warrant interventions that address both systemic inequities and provider-level challenges. In multi-team settings such as in breast reconstruction, where oncologists, breast surgeons, and plastic surgeons collaboratively shape care decisions, clear and effective communication is crucial for ensuring timely care [61]. Accordingly, standardized referral protocols should be implemented to streamline communication [58]. While this standardization should help reduce provider bias [61], provider-level influences can be further addressed by integrating implicit bias training into surgical education [62]. Patient education, delivered through both conversation and written materials, is also essential for facilitating shared decision-making and ensuring timely care [63]. Moreover, early involvement of social work can help identify and overcome logistical barriers [64].

Notably, many institutions have already begun implementing strategies aligned with these recommendations, reflecting a broader shift toward equity-focused care delivery [65]. Increasingly, hospitals are collecting detailed demographic and social determinant data to better identify disparities, embedding patient navigators and community health workers to guide patients through complex care pathways, and aligning performance metrics with equity goals [65]. Health systems are also leveraging telehealth, transportation support, and flexible scheduling to reduce geographic and logistical barriers, particularly for underserved populations [65]. These efforts, coupled with targeted community partnerships and ongoing workforce training in cultural competence and bias mitigation, should help bridge gaps in access and timeliness of care within single-institution settings.

Although the internal consistency of this study and alignment of results with the existing literature lend credibility to these recommendations, several limitations merit consideration. As a retrospective analysis, it is subject to potential selection bias and confounding factors that were not accounted for in the adjusted models. Additionally, the study was conducted at a single institution, which may limit the generalizability of findings to broader populations. Future research should focus on multi-institutional analyses to validate these findings across different healthcare settings. While “Other” and “unknown” racial groups stood as a proxy for Hispanic-identifying patients in the present study, future research should also specifically address ethnicity-based care delays.

## 5. Conclusions

The present study revealed significant racial disparities in the timeliness of breast reconstructive care, with all non-White patient groups, and Black patients in particular, experiencing prolonged intervals between breast cancer diagnosis and key milestones in the reconstructive process, including PRS consultation, initiation, and completion of reconstruction. These inequities persist even after adjusting for confounding factors, including insurance coverage. When considered alongside the existing literature on racial disparities, these findings underscore the influence of systemic barriers, such as logistical challenges and provider bias, in perpetuating inequitable access to timely reconstructive care.

## Figures and Tables

**Table 1:** Baseline characteristics across self-identified racial groups.

	White	Black	Asian	Other	Unknown	*p*-Value
	*n* = 745	*n* = 337	*n* = 199	*n* = 278	*n* = 103	
Age	53 (12)	54 (11)	48 (10)	53 (11)	52 (11)	**<0.001**
BMI	26.8 (7.9)	30.2 (5.8)	24.6 (4.3)	28.7 (5.4)	28.3 (5.3)	**<0.001**
Ethnicity						**<0.001**
Latino	16 (2.1%)	6 (1.8%)	1 (0.5%)	138 (50%)	77 (75%)	
Non-Latino	723 (97%)	331 (98%)	198 (99%)	130 (47%)	23 (22%)	
Comorbidities						
CAD	24 (3.2%)	20 (5.9%)	2 (1.0%)	10 (3.6%)	3 (2.9%)	**0.0454**
MI	4 (0.5%)	5 (1.5%)	1 (0.5%)	2 (0.7%)	1 (1.0%)	0.4944
Heart Failure	42 (5.6%)	34 (10%)	12 (6.0%)	18 (6.5%)	15 (15%)	**0.0033**
PAD	19 (2.6%)	16 (4.7%)	2 (1.0%)	14 (5.0%)	5 (4.9%)	**0.0284**
CVA	14 (1.9%)	13 (3.9%)	3 (1.5%)	5 (1.8%)	4 (3.9%)	0.1984
Diabetes	33 (4.4%)	40 (12%)	11 (5.5%)	45 (16%)	11 (11%)	**<0.001**
HTN	167 (22%)	148 (44%)	33 (17%)	98 (35%)	28 (27%)	**<0.001**
HLD	116 (16%)	83 (25%)	28 (14%)	72 (26%)	18 (17%)	**<0.001**
Asthma	64 (8.6%)	43 (13%)	7 (3.5%)	41 (15%)	13 (13%)	**<0.001**
COPD	20 (2.7%)	9 (2.7%)	0 (0%)	7 (2.5%)	2 (1.9%)	0.1144
Liver Disease	37 (5.0%)	25 (7.4%)	20 (10%)	15 (5.4%)	7 (6.8%)	0.0863
Renal History	7 (0.9%)	20 (6.0%)	3 (1.5%)	8 (2.9%)	2 (1.9%)	**<0.001**
Reconstruction Type						**<0.001**
Immediate Implant	58 (7.8%)	16 (4.7%)	8 (4.0%)	15 (5.4%)	1 (1.0%)	
Delayed Implant	349 (47%)	96 (28%)	64 (32%)	106 (38%)	6 (5.8%)	
Immediate Autologous	270 (36%)	172 (51%)	112 (56%)	119 (43%)	82 (80%)	
Delayed Autologous	44 (5.9%)	37 (11%)	11 (5.5%)	31 (11%)	13 (13%)	
Other	24 (3.2%)	16 (4.7%)	4 (2.0%)	7 (2.5%)	1 (1.0%)	
Laterality						**<0.001**
Unilateral	251 (34%)	181 (54%)	118 (59%)	137 (49%)	53 (51%)	
Bilateral	494 (66%)	156 (46%)	81 (41%)	141 (51%)	50 (49%)	
Insurance						**<0.001**
Medicare	157 (21%)	103 (31%)	21 (11%)	71 (26%)	15 (15%)	
Medicaid	76 (10%)	59 (18%)	49 (25%)	81 (29%)	30 (29%)	
Private	503 (68%)	175 (52%)	125 (63%)	126 (45%)	56 (54%)	
Procedure Time						
Index Procedure	5.06 (2.54)	5.60 (2.94)	5.42 (2.98)	5.55 (2.84)	7.18 (2.70)	**<0.001**
Aggregate Procedures	6.16 (2.47)	6.57 (2.95)	6.31 (3.00)	6.77 (2.71)	7.94 (2.65)	**<0.001**
Clinical Timelines						
Diagnosis to PRS consult	80 (233)	94 (176)	346 (3243)	110 (267)	85 (132)	**<0.001**
Diagnosis to Index Procedure	261 (2447)	150 (210)	382 (3174)	160 (296)	138 (153)	**<0.001**
Diagnosis to Final Procedure	390 (2528)	280 (372)	473 (3209)	289 (449)	224 (353)	**0.0172**

As shown in Table 1, Latino ethnicity was most common in the “Other” (50%) and “Unknown” (75%) race categories. Black patients had the highest BMI and the highest comorbidity burden. Medicare coverage was most common among Black patients (31%), Medicaid among “Other” race patients (29%), and private insurance among White patients (68%).

**Table 2:** Univariable regression of race on clinical timelines ^1^.

	Diagnosis to PRS Consult		Diagnosis to Index Procedure		Diagnosis to Final Procedure	
	Beta (95% CI) ^1^	*p*-Value	Beta (95% CI) ^1^	*p*-Value	Beta (95% CI) ^1^	*p*-Value
Overall						
Black	0.339 (0.162 to 0.516)	**<0.001**	0.302 (0.168 to 0.435)	**<0.001**	0.083 (−0.073 to 0.239)	0.3
Asian	0.300 (0.083 to 0.517)	**0.007**	0.164 (0.001 to 0.326)	**0.049**	−0.033 (−0.219 to 0.153)	0.73
Other	0.380 (0.188 to 0.571)	**<0.001**	0.308 (0.164 to 0.451)	**<0.001**	0.172 (0.005 to 0.339)	**0.044**
Unknown	0.296 (0.011 to 0.582)	**0.042**	0.297 (0.080 to 0.514)	**0.007**	−0.192 (−0.436 to 0.051)	0.12
ABR Cohort						
Black	0.238 (0.009 to 0.468)	**0.042**	0.142 (−0.031 to 0.315)	0.11	0.201 (−0.002 to 0.405)	0.053
Asian	0.320 (0.041 to 0.599)	**0.025**	0.117 (−0.092 to 0.326)	0.27	0.042 (−0.204 to 0.288)	0.74
Other	0.245 (−0.020 to 0.510)	0.07	0.197 (0.003 to 0.392)	**0.047**	0.206 (−0.023 to 0.435)	0.078
Unknown	0.261 (−0.042 to 0.565)	0.091	0.213 (−0.016 to 0.442)	0.068	0.185 (−0.082 to 0.452)	0.17
IBR Cohort						
Black	0.537 (0.242 to 0.831)	**<0.001**	0.516 (0.298 to 0.734)	**<0.001**	0.342 (0.119 to 0.565)	**0.003**
Asian	0.259 (−0.088 to 0.607)	0.14	0.192 (−0.067 to 0.451)	0.15	0.150 (−0.095 to 0.396)	0.23
Other	0.505 (0.226 to 0.785)	**<0.001**	0.391 (0.180 to 0.603)	**<0.001**	0.254 (0.046 to 0.462)	**0.017**
Unknown	0.178 (−1.071 to 1.426)	0.78	0.243 (−0.725 to 1.211)	0.62	0.097 (−0.792 to 0.987)	0.83

^1^ Relative to patients identifying as White.

**Table 3:** Multivariable regression of race on clinical timelines ^1,2^.

	Diagnosis to PRS Consult		Diagnosis to Index Procedure		Diagnosis to Final Procedure	
	Beta (95% CI) ^1^	*p*-Value	Beta (95% CI) ^1^	*p*-Value	Beta (95% CI) ^1^	*p*-Value
Overall						
Black	0.307 (0.121 to 0.493)	**0.001**	0.254 (0.117 to 0.390)	**<0.001**	0.213 (0.084 to 0.342)	**0.001**
Asian	0.267 (0.042 to 0.491)	**0.02**	0.157 (−0.008 to 0.321)	0.062	0.091 (−0.061 to 0.243)	0.24
Other	0.332 (0.095 to 0.568)	**0.006**	0.283 (0.111 to 0.456)	**0.001**	0.120 (−0.043 to 0.283)	0.15
Unknown	0.276 (−0.082 to 0.634)	0.13	0.277 (0.011 to 0.544)	**0.042**	0.147 (−0.098 to 0.391)	0.24
ABR Cohort						
Black	0.256 (0.015 to 0.496)	**0.037**	0.149 (−0.026 to 0.324)	0.1	0.155 (−0.019 to 0.328)	0.08
Asian	0.299 (0.005 to 0.593)	**0.046**	0.152 (−0.061 to 0.364)	0.16	0.128 (−0.082 to 0.337)	0.23
Other	0.194 (−0.118 to 0.506)	0.22	0.153 (−0.067 to 0.373)	0.17	0.047 (−0.171 to 0.264)	0.68
Unknown	0.237 (−0.175 to 0.650)	0.26	0.197 (−0.102 to 0.496)	0.2	0.130 (−0.161 to 0.421)	0.38
IBR Cohort						
Black	0.459 (0.148 to 0.770)	**0.004**	0.447 (0.218 to 0.675)	**<0.001**	0.322 (0.120 to 0.523)	**0.002**
Asian	0.224 (−0.130 to 0.578)	0.21	0.172 (−0.091 to 0.436)	0.2	0.044 (−0.174 to 0.263)	0.69
Other	0.521 (0.141 to 0.900)	**0.007**	0.411 (0.124 to 0.698)	**0.005**	0.155 (−0.099 to 0.410)	0.23
Unknown	0.186 (−1.121 to 1.494)	0.78	0.233 (−0.779 to 1.245)	0.65	−0.139 (−0.959 to 0.681)	0.74

^1^ Relative to patients identifying as White. ^2^ Controlling for age, BMI, comorbidities, insurance, reconstruction type and laterality.

## Data Availability

The data presented in this study are available on request from the corresponding author due to privacy and ethical restrictions.

## Abbreviations

The following abbreviations are used in this manuscript:

PRS	Plastic and Reconstructive Surgery
CAD	Coronary Artery Disease
HTN	Hypertension
HLD	Hyperlipidemia
MI	Myocardial Infarction
PAD	Peripheral Artery Disease
CVA	Cerebrovascular Accidents
HF	Heart Failure
COPD	Chronic Obstructive Pulmonary Disease
ABR	Autologous Breast Reconstruction
IBR	Implant-Based Reconstruction
SD	Standard Deviation
CI	Confidence Interval

## References

[1] Chen J., Vargas-Bustamante A., Mortensen K., Ortega A.N. (2016). Racial and Ethnic Disparities in Health Care Access and Utilization Under the Affordable Care Act. Med. Care.

[2] Chou C.-H., Tulolo A., Raver E.W., Hsu C.-H., Young G. (2013). Effect of Race and Health Insurance on Health Disparities: Results from the National Health Interview Survey 2010. J. Health Care Poor Underserved.

[3] Monheit A.C., Vistnes J.P. (2000). Race/Ethnicity and Health Insurance Status: 1987 and 1996. Med. Care Res. Rev..

[4] Mahajan S., Caraballo C., Lu Y., Valero-Elizondo J., Massey D., Annapureddy A.R., Roy B., Riley C., Murugiah K., Onuma O. (2021). Trends in Differences in Health Status and Health Care Access and Affordability by Race and Ethnicity in the United States, 1999–2018. J. Am. Med. Assoc..

[5] Koh H.K., Graham G., Glied S.A. (2011). Reducing Racial And Ethnic Disparities: The Action Plan From The Department of Health And Human Services. Health Aff..

[6] Du X.L., Liu C.-C. (2010). Racial/Ethnic Disparities in Socioeconomic Status, Diagnosis, Treatment and Survival among Medicare-insured Men and Women with Head and Neck Cancer. J. Health Care Poor Underserved.

[7] Dunlop D.D., Manheim L.M., Song J., Sohn M.-W., Feinglass J.M., Chang H.J., Chang R.W. (2008). Age and Racial/Ethnic Disparities in Arthritis-Related Hip and Knee Surgeries. Med. Care.

[8] Fairfield K.M., Lucas F.L., Earle C.C., Small L., Trimble E.L., Warren J.L. (2010). Regional variation in cancer-directed surgery and mortality among women with epithelial ovarian cancer in the medicare population. Cancer.

[9] Farjah F., Wood D.E., Yanez N.D., Vaughan T.L., Symons R.G., Krishnadasan B., Flum D.R. (2009). Racial Disparities Among Patients With Lung Cancer Who Were Recommended Operative Therapy. Arch. Surg..

[10] Gundle K.R., McGlaston T.J., Ramappa A.J. (2010). Effect of Insurance Status on the Rate of Surgery Following a Meniscal Tear. J. Bone Jt. Surg..

[11] Halm E.A., Tuhrim S., Wang J.J., Rojas M., Rockman C., Riles T.S., Chassin M.R. (2009). Racial and Ethnic Disparities in Outcomes and Appropriateness of Carotid Endarterectomy. Stroke.

[12] Hanchate A.D., Zhang Y.D., Felson D.T., Ash A.S. (2008). Exploring the Determinants of Racial and Ethnic Disparities in Total Knee Arthroplasty. Med. Care.

[13] Haas J.S., Brawarsky P., Iyer A., Fitzmaurice G.M., Neville B.A., Earle C. (2011). Association of area sociodemographic characteristics and capacity for treatment with disparities in colorectal cancer care and mortality. Cancer.

[14] Kruper L., Holt A., Xu X.X., Duan L., Henderson K., Bernstein L., Ellenhorn J. (2011). Disparities in Reconstruction Rates After Mastectomy: Patterns of Care and Factors Associated with the Use of Breast Reconstruction in Southern California. Ann. Surg. Oncol..

[15] Murphy M.M., Simons J.P., Ng S.C., McDade T.P., Smith J.K., Shah S.A., Zhou Z., Earle C.C., Tseng J.F. (2009). Racial Differences in Cancer Specialist Consultation, Treatment, and Outcomes for Locoregional Pancreatic Adenocarcinoma. Ann. Surg. Oncol..

[16] Osborne N.H., Mathur A.K., Upchurch G.R., Dimick J.B. (2010). Understanding the Racial Disparity in the Receipt of Endovascular Abdominal Aortic Aneurysm Repair. Arch. Surg..

[17] Steyerberg E.W., Earle C.C., Neville B.A., Weeks J.C. (2005). Racial Differences in Surgical Evaluation, Treatment, and Outcome of Locoregional Esophageal Cancer: A Population-Based Analysis of Elderly Patients. J. Clin. Oncol..

[18] Trinh Q., Schmitges J., Sun M., Sukumar S., Sammon J., Shariat S.F., Jeldres C., Bianchi M., Tian Z., Perrotte P. (2011). Improvement of racial disparities with respect to the utilization of minimally invasive radical prostatectomy in the United States. Cancer.

[19] Yang R., Cheung M.C., Byrne M.M., Huang Y., Nguyen D., Lally B.E., Koniaris L.G. (2010). Do racial or socioeconomic disparities exist in lung cancer treatment?. Cancer.

[20] Vogel T.R., Cantor J.C., Dombrovskiy V.Y., Haser P.B., Graham A.M. (2008). AAA Repair: Sociodemographic Disparities in Management and Outcomes. Vasc. Endovasc. Surg..

[21] Bryant C.S., Kumar S., Shah J.P., Mahdi H., Ali-Fehmi R., Munkarah A.R., Deppe G., Morris R.T. (2009). Racial disparities in survival among patients with germ cell tumors of the ovary—United States. Gynecol. Oncol..

[22] Al-Refaie W.B., Muluneh B., Zhong W., Parsons H.M., Tuttle T.M., Vickers S.M., Habermann E.B. (2012). Who Receives Their Complex Cancer Surgery at Low-Volume Hospitals?. J. Am. Coll. Surg..

[23] Neighbors C.J.P., Rogers M.L., Shenassa E.D.S., Sciamanna C.N., Clark M.A., Novak S.P. (2007). Ethnic/Racial Disparities in Hospital Procedure Volume for Lung Resection for Lung Cancer. Med. Care.

[24] Aranda M.A., McGory M., Sekeris E., Maggard M., Ko C., Zingmond D.S. (2008). Do racial/ethnic disparities exist in the utilization of high-volume surgeons for women with ovarian cancer?. Gynecol. Oncol..

[25] Pollack C.E., Bekelman J.E., Epstein A.J., Liao K., Wong Y.-N., Armstrong K. (2011). Racial Disparities in Changing to a High-volume Urologist Among Men With Localized Prostate Cancer. Med. Care.

[26] Castellanos L.R., Normand S.-L.T., Ayanian J.Z. (2009). Racial and Ethnic Disparities in Access to Higher and Lower Quality Cardiac Surgeons for Coronary Artery Bypass Grafting. Am. J. Cardiol..

[27] Anger J.T., Rodríguez L.V., Wang Q., Chen E., Pashos C.L., Litwin M.S. (2007). Racial Disparities in the Surgical Management of Stress Incontinence Among Female Medicare Beneficiaries. J. Urol..

[28] Parsons H.M., Habermann E.B., Stain S.C., Vickers S.M., Al-Refaie W.B. (2012). What Happens to Racial and Ethnic Minorities after Cancer Surgery at American College of Surgeons National Surgical Quality Improvement Program Hospitals?. J. Am. Coll. Surg..

[29] Artinyan A., Mailey B., Sanchez-Luege N., Khalili J., Sun C., Bhatia S., Wagman L.D., Nissen N., Colquhoun S.D., Kim J. (2010). Race, ethnicity, and socioeconomic status influence the survival of patients with hepatocellular carcinoma in the United States. Cancer.

[30] Kelz R.R., Gimotty P.A., Polsky D., Norman S., Fraker D., DeMichele A. (2004). Morbidity and mortality of colorectal carcinoma surgery differs by insurance status. Cancer.

[31] Kennedy B.S., Fortmann S.P., Stafford R.S. (2007). Elective and isolated carotid endarterectomy: Health disparities in utilization and outcomes, but not readmission. J. Natl. Med. Assoc..

[32] Kim J., Artinyan A., Mailey B., Christopher S., Lee W., McKenzie S., Chen S.L., Bhatia S., Pigazzi A., Garcia-Aguilar J. (2011). An Interaction of Race and Ethnicity With Socioeconomic Status in Rectal Cancer Outcomes. Ann. Surg..

[33] Konety S.H., Sarrazin M.S.V., Rosenthal G.E. (2005). Patient and Hospital Differences Underlying Racial Variation in Outcomes After Coronary Artery Bypass Graft Surgery. Circulation.

[34] Lemaire A., Cook C., Tackett S., Mendes D.M., Shortell C.K. (2008). The impact of race and insurance type on the outcome of endovascular abdominal aortic aneurysm (AAA) repair. J. Vasc. Surg..

[35] Lucas F.L., Stukel T.A., Morris A.M., Siewers A.E., Birkmeyer J.D. (2006). Race and Surgical Mortality in the United States. Ann. Surg..

[36] Nathan H., Frederick W., Choti M.A., Schulick R.D., Pawlik T.M. (2008). Racial Disparity in Surgical Mortality after Major Hepatectomy. J. Am. Coll. Surg..

[37] Osborne N.H., Upchurch G.R., Mathur A.K., Dimick J.B. (2009). Explaining racial disparities in mortality after abdominal aortic aneurysm repair. J. Vasc. Surg..

[38] Ricciardi R., Selker H.P., Baxter N.N., Marcello P.W., Roberts P.L., Virnig B.A. (2008). Disparate use of minimally invasive surgery in benign surgical conditions. Surg. Endosc..

[39] Sosa J.A., Mehta P.J., Wang T.S., Yeo H.L., Roman S.A. (2007). Racial Disparities in Clinical and Economic Outcomes From Thyroidectomy. Ann. Surg..

[40] Singh T.P., Almond C., Givertz M.M., Piercey G., Gauvreau K. (2011). Improved Survival in Heart Transplant Recipients in the United States. Circ. Heart Fail..

[41] Azin A., Hirpara D.H., Doshi S.M., Chesney T.R.M., Quereshy F.A.M., Chadi S.A.M. (2020). Racial Disparities in Surgery. Ann. Surg. Open.

[42] Morris A.M., Wei Y., Birkmeyer N.J., Birkmeyer J.D. (2006). Racial Disparities in Late Survival after Rectal Cancer Surgery. J. Am. Coll. Surg..

[43] Rangrass G., Ghaferi A.A., Dimick J.B. (2014). Explaining Racial Disparities in Outcomes After Cardiac Surgery. J. Am. Med. Assoc. Surg..

[44] Sarver M.M., Rames J.D., Ren Y., Greenup R.A., Shammas R.L., Hwang E.S., Hollenbeck S.T.M., Hyslop T., Butler P.D.M., Fayanju O.M. (2022). Racial and Ethnic Disparities in Surgical Outcomes after Postmastectomy Breast Reconstruction. J. Am. Coll. Surg..

[45] Berlin N.L., Wilkins E.G., Alderman A.K. (2018). Addressing Continued Disparities in Access to Breast Reconstruction on the 20th Anniversary of the Women’s Health and Cancer Rights Act. J. Am. Med. Assoc. Surg..

[46] Doren E.L., Park K., Olson J. (2023). Racial disparities in postmastectomy breast reconstruction following implementation of the affordable care act: A systematic review using a minority health and disparities research framework. Am. J. Surg..

[47] Connors S.K., Goodman M.S., Myckatyn T., Margenthaler J., Gehlert S. (2021). Racial Disparities in Breast Reconstruction at a Comprehensive Cancer Center. J. Racial Ethn. Health Dispar..

[48] Epstein S., Tran B.N., Cohen J.B., Lin S.J., Singhal D., Lee B.T. (2018). Racial disparities in postmastectomy breast reconstruction: National trends in utilization from 2005 to 2014. Cancer.

[49] Butler P.D., Nelson J.A., Fischer J.P., Wink J.D., Chang B., Fosnot J., Wu L.C., Serletti J.M. (2016). Racial and age disparities persist in immediate breast reconstruction: An updated analysis of 48,564 patients from the 2005 to 2011 American College of Surgeons National Surgery Quality Improvement Program data sets. Am. J. Surg..

[50] Mahmoudi E., Lu Y., Metz A.K., Momoh A.O., Chung K.C. (2017). Association of a Policy Mandating Physician-Patient Communication With Racial/Ethnic Disparities in Postmastectomy Breast Reconstruction. J. Am. Med. Assoc. Surg..

[51] Zhong T., McCarthy C., Min S., Zhang J., Beber B., Pusic A.L., Hofer S.O.P. (2011). Patient satisfaction and health-related quality of life after autologous tissue breast reconstruction. Cancer.

[52] Harris D.R., Andrews R., Elixhauser A. (1997). Racial and gender differences in use of procedures for black and white hospitalized adults. Ethn. Dis..

[53] Williams D.R., Rucker T.D. (2000). Understanding and Addressing Racial Disparities in Health Care. Health Care Financ. Rev..

[54] Hernandez S.M., Sparks P.J. (2020). Barriers to Health Care Among Adults With Minoritized Identities in the United States, 2013–2017. Am. J. Public Health.

[55] Syed S.T., Gerber B.S., Sharp L.K. (2013). Traveling towards Disease: Transportation Barriers to Health Care Access. J. Community Health.

[56] Kullgren J.T., McLaughlin C.G., Mitra N., Armstrong K. (2011). Nonfinancial Barriers and Access to Care for U.S. Adults. Health Serv. Res..

[57] Primm K., Muraleetharan D., Gilreath T. (2019). Use of Emergency Departments for Preventative Care Among Adults in the United States: Estimates From the 2017 National Health Interview Survey. J. Emerg. Med..

[58] Cheung P.T., Wiler J.L., Lowe R.A., Ginde A.A. (2012). National Study of Barriers to Timely Primary Care and Emergency Department Utilization Among Medicaid Beneficiaries. Ann. Emerg. Med..

[59] Sabin J.A.P., Rivara F.P., Greenwald A.G. (2008). Physician Implicit Attitudes and Stereotypes About Race and Quality of Medical Care. Med. Care.

[60] van Ryn M. (2002). Research on the Provider Contribution to Race/Ethnicity Disparities in Medical Care. Med. Care.

[61] Greenwood-Lee J., Jewett L., Woodhouse L., Marshall D.A. (2018). A categorisation of problems and solutions to improve patient referrals from primary to specialty care. BMC Health Serv. Res..

[62] Chen R.P., Tang J., Weller L.N.H., Boscardin C.K., Ehie O.A. (2024). The Impact of an Interactive Unconscious Bias Training on Perioperative Learners. J. Educ. Perioper. Med..

[63] Bhattad P.B., Pacifico L. (2022). Empowering Patients: Promoting Patient Education and Health Literacy. Cureus.

[64] Kreuter M.W., Thompson T., McQueen A., Garg R. (2021). Addressing Social Needs in Health Care Settings: Evidence, Challenges, and Opportunities for Public Health. Annu. Rev. Public Health.

[65] Chisolm D.J., Dugan J.A., Figueroa J.F., Lane-Fall M.B., Roby D.H., Rodriguez H.P., Ortega A.N. (2023). Improving health equity through health care systems research. Health Serv. Res..

